# Serum Metabolic Profiles of Chinese Women With Perimenopausal Obesity Explored by the Untargeted Metabolomics Approach

**DOI:** 10.3389/fendo.2021.637317

**Published:** 2021-09-24

**Authors:** Shanshan Ding, Mingyi Chen, Ying Liao, Qiliang Chen, Xuejuan Lin, Shujiao Chen, Yujuan Chai, Candong Li, Tetsuya Asakawa

**Affiliations:** ^1^ Research Base of Traditional Chinese Medicine Syndrome, Fujian University of Traditional Chinese Medicine, Fuzhou, China; ^2^ School of Basic Medicine, Guangzhou University of Chinese Medicine, Guangzhou, China; ^3^ School of Medical Engineering, Health Science Center, Shenzhen University, Shenzhen, China; ^4^ Department of Neurosurgery, Hamamatsu University School of Medicine, Hamamatsu, Japan; ^5^ Department of Neurology, The Eighth Affiliated Hospital, Sun Yat-Sen University, Shenzhen, China

**Keywords:** perimenopausal obesity, metabolic profiles, the ultra-high performance liquid chromatography-quadrupole time-of-flight/mass spectrometry, metabolic pathway, metabolomics

## Abstract

By far, no study has focused on observing the metabolomic profiles in perimenopause-related obesity. This study attempted to identify the metabolic characteristics of subjects with perimenopause obesity (PO). Thirty-nine perimenopausal Chinese women, 21 with PO and 18 without obesity (PN), were recruited in this study. A conventional ultra-high-performance liquid chromatography-quadrupole time-of-flight/mass spectrometry (UHPLC-QTOF/MS) followed by principal component analysis (PCA) and orthogonal partial least-squares discriminant analysis (OPLS-DA) were used as untargeted metabolomics approaches to explore the serum metabolic profiles. Kyoto Encyclopedia of Genes and Genomes (KEGG) and MetaboAnalyst were used to identify the related metabolic pathways. A total of 46 differential metabolites, along with seven metabolic pathways relevant to PO were identified, which belonged to lipid, amino acids, carbohydrates, and organic acids. As for amino acids, we found a significant increase in l-arginine and d-ornithine in the positive ion (POS) mode and l-leucine, l-valine, l-tyrosine, and *N*-acetyl-l-tyrosine in the negative ion (NEG) mode and a significant decrease in l-proline in the POS mode of the PO group. We also found phosphatidylcholine (PC) (16:0/16:0), palmitic acid, and myristic acid, which are associated with the significant upregulation of lipid metabolism. Moreover, the serum indole lactic acid and indoleacetic acid were upregulated in the NEG mode. With respect to the metabolic pathways, the d-arginine and d-ornithine metabolisms and the arginine and proline metabolism pathways in POS mode were the most dominant PO-related pathways. The changes of metabolisms of lipid, amino acids, and indoleacetic acid provided a pathophysiological scenario for Chinese women with PO. We believe that the findings of this study are helpful for clinicians to take measures to prevent the women with PO from developing severe incurable obesity-related complications, such as cardiovascular disease and stroke.

## Introduction

Perimenopause is a peculiar period before and after menopause in women (approximately 40–55 years). Under the background of weakening ovarian function and declining estrogen level, a battery of physiological and pathological changes may happen. Many women experience various symptoms, such as night sweats, hot flushes, mood swings, menstrual disorder, and vaginal dryness (1). Moreover, perimenopause is closely associated with many diseases, such as metabolic syndrome (MetS) (2), breast cancer (3), osteoporosis (4), and cardiovascular disease (CVD) (5). Of those, perimenopause obesity (PO) is a growing concern because it might result in a spectrum of obesity-related diseases. The pathophysiological state of PO is quite complicated. It is influenced by many pathogenic factors, of which estrogen deficiency plays a key role (6). The complicated interactions among these factors sometimes make the situation intractable. Estrogen deficiency has been known to cause fat accumulation and increase body weight (BW) (7), which are highly associated with MetS, type 2 diabetes (T2D), CVD, and stroke. Thus, clarifying the metabolic characteristics in PO is extremely helpful because understanding the pathophysiological mechanisms and the affecting factors relevant to PO is useful, which contribute to the selection of an appropriate clinical strategy to prevent perimenopause women from progressing to intractable complications.

Alternatively, metabolomics has been developed as a promising technology in achieving the quantitative measurement of dynamic metabolomic profiles of a living subject in a certain pathophysiological state. The commonly used methods for sample analysis are mass spectrometry (MS) or ^1^H nuclear magnetic resonance (NMR). The main strength of metabolomics approach is that it can conduct simultaneous measurements of many small molecular metabolites (8, 9). It is also effective for diseases with a complicated metabolic response (9, 10). Hence, it has been widely used for early diagnosis, exploring novel biomarkers and treatment targets, and elucidating the mechanisms of certain diseases, particularly for metabolic diseases, such as T2D, MetS, and obesity. Recently, we performed a ^1^H NMR study to explore the metabolic characteristics of untreated patients with MetS. We found that such untreated patients have peculiar metabolic characteristics, such as ketosis tendency, early kidney damage, activation of oxidative signaling, and inflammatory response, which suggested that the pathophysiological state might worsen if MetS is left untreated (11). The metabolomics approach is also used for metabolomic profile elucidation in obesity. Chen et al. used liquid chromatography/time-of-flight MS (LC–MS) and gas chromatography/quadrupole mass spectrometry (GC-MS) methods to explore the metabolic characteristics in patients with metabolic abnormal obesity and healthy obesity. They found that the metabolic characteristics were different between metabolic abnormal obesity and metabolic healthy obesity. A battery of metabolites, such as l-kynurenine, glycerol 1-phosphate, glycolic acid, tagatose, methyl palmitate, and uric acid, are useful in distinguishing these different types of obesity (12). Another study investigated the relationship between metabolomic profiles and clinical indices in obese children using a ^1^H NMR technology. Saner et al. found that the body mass index (BMI) and phenylalanine, total body fat % and lipids in medium high-density lipoprotein (HDL), and waist circumference and tyrosine were positively correlated, whereas total body fat % and the ratio of docosahexaenoic acid/total fatty acids and histidine were negatively correlated (13). Rangel-Huerta et al. conducted a systematic review summarizing previous studies using the metabolomics approach to investigate metabolomic profiles in subjects with obesity or obesity-related metabolic changes. They analyzed 33 included literatures and found that these studies could be classified into four types: (1) investigation of metabolic characteristics in an obese subject, (2) comparing the difference of the response to dietary challenges between the obese and nonobese population, (3) using metabolomics to predict weight loss and other interventions, and (4) metabolomic profiles in different dietary patterns (14). Albeit these previous studies investigated the obesity-related metabolomic profiles from different perspectives or in different subjects, they all endorsed that the metabolomics approach is a powerful tool in obesity researches, such as identifying the metabolic characteristics associated with obesity, exploring novel biomarkers/targets for obesity, or observing changes of metabolites induced by interventions against obesity. However, to our knowledge, there is no research that particularly observed the metabolomic profiles in perimenopause-related obesity. This study attempted to identify the metabolic characteristics in subjects with PO. We believe that the findings of this study contribute to a better understanding of the pathophysiological nature of PO, which is beneficial for exploring effective interventions against PO to prevent obesity-related severe complications in perimenopausal women.

## Methods and Materials

### Participants

Participants were recruited from the Third Affiliated Hospital of Fujian Traditional Chinese Medical University between October 2016 and March 2018. A total of 39 perimenopausal women were enrolled based on voluntary participation, including 18 without obesity (PN) and 21 with obesity (PO). Inclusion criteria were listed as follows: (1) perimenopause, women aged 40 to 55 years with menstrual disorders or amenorrhea for ≥3 and <12 months (15), and (2) simple obesity (we used the Guidelines for Prevention and Control of Overweight and Obesity in Chinese Adults) (16). In brief, waist circumference (WC) ≥ 80 cm or BMI ≥ 28 kg/m^2^ were included. Exclusion criteria were: (1) type 1 diabetes, gestational diabetes, secondary hypertension, or hyperlipemia; (2) serious heart, liver, kidney, or other complications; (3) psychiatric disorders; (4) secondary or drug-induced obesity; and (5) pituitary tumors or Cushing syndrome. This study was designed and conducted according to the *Declaration of Helsinki of the World Medical Association (2000)* and was approved and supervised by the Ethics Committee of the Fujian University of Traditional Chinese Medicine (approval number: SQ2014-007-01, study period from 2014 to 2019). The investigation protocol was explained in detail to all participants and their relatives. Informed consent was obtained from each participant before the study initiation.

### Clinical Indices and Serum Sample Collection

Clinical indices of participants in a fasting state, including height, BW, WC, serum triglyceride (TG), total cholesterol (TC), high-density lipoprotein cholesterol (HDL-C), and low-density lipoprotein cholesterol (LDL-C), were examined. BMI was calculated as BW/height^2^ (kg/m^2^). WC was measured with tape as the circumference of the horizontal edge of the midpoint at the lower edge of the costal arch. Elbow venous blood was collected in a fasting state from 8:00 am to 9:00 am. For menstruating women, blood samples were collected on the third to fifth day after menstruation. For women whose menstruation had stopped for ≥6 months, it was collected on any day at their convenience. These (5 ml) were collected and centrifuged for 15 min at 3,500 rpm and then were preserved at −80°C for subsequent experiments. Because the estrogen level may be one of the factors affecting the results, the serum estrogen levels were measured using a standard enzyme-linked immunosorbent assay, which was as described in our previous study (6).

### Sample Preparation

The serum samples were prepared as per the following procedure introduced in a previous study (17). Briefly, first, the extraction solvent (400 μl, V methanol: V acetonitrile = 1:1) was added to a 100-μl sample, mixed by vortex for 30 s, and treated with sonicate for 10 min in an ice-water bath. The solution was then incubated at −20°C for 1 h and centrifuged at 4°C and 12,000 rpm for 15 min to precipitate the proteins for the aim of preventing the macromolecules from blocking the chromatographic column and electrospray ionization probe. Subsequently, 420 µl of the supernatant was transferred into EP tubes, and the extracts were dried in a vacuum concentrator without heating and then added into a 100-μl extraction liquid (V acetonitrile: V water = 1:1) reconstitution, followed by vortexing for 30 s and sonicating for 10 min (4°C water bath), and centrifuged for 15 min at 12,000 rpm, 4°C. The supernatant was transferred into a fresh 2-ml LC/MS glass vial pending for detection. Equal volumes (10 μl) of supernatant from different individual serum samples were pooled as the quality control (QC) sample for the UHPLC-QTOF/MS analysis. The ionization source of LC–MS is electrospray ionization, including positive ion (POS) and negative ion (NEG) modes. The QC samples were used to assess the reproducibility and reliability of the LC–MS system.

### UHPLC-QTOF/MS Conditions

Serum metabolic profiling analysis was performed using a 1290 UHPLC system (Agilent Technologies, Santa Clara, CA, USA) with a Waters UPLC BEH Amide column (1.7 μm; 2.1 × 100 mm) coupled to Triple TOF 6600 (AB Sciex, Framingham, MA, USA) & QTOF 6550 (Agilent) according to a previous study (18). The aim of using the two instruments here was to improve the quality and reliability of the experiments. The mobile phase consisting of 25 mM CH_3_COONH_4_ and 25 mM NH_4_OH in water (pH = 9.75) (A), along with acetonitrile (B), was performed with elution gradient as follows: 0 min, 95% B; 7 min, 65% B; 9 min, 40% B; 9.1 min, 95% B; and 12 min, 95% B at 0.5 ml/min. The injection volume was 2 μl. A Triple TOF mass spectrometer was used for its ability to acquire MS/MS spectra on an information-dependent basis (IDA) during the LC/MS experiment. In this mode, Analyst TF 1.7 software (AB Sciex) collected and triggered the acquisition of MS/MS spectra under the preselected conditions while it continuously evaluated the acquired data. In each cycle, 12 precursor ions, which have a >100 intensity, were chosen for fragmentation at a collision energy of 30 V (15 MS/MS events with product ion accumulation time of 50 msec each). Electrospray ionization (ESI) source conditions were set as follows: ion source gas 1 as 60 Psi, ion source gas 2 as 60 Psi, curtain gas as 35 Psi, source temperature 650°C, and IonSpray Voltage Floating (ISVF) 5000 V or –4000 V in positive or negative modes, respectively (19). The UHPLC-QTOF/MS program was run in POS mode then in NEG mode.

### UHPLC-QTOF/MS Data Processing and Statistical Analysis

MS raw data were converted to the mzXML format using the ProteoWizard software and processed with R package XCMS (version 3.2). The preprocessing results generated a data matrix that consisted of the retention time (RT), mass-to-charge ratio (m/z) values, and peak intensity. R package CAMERA was used for the peak annotation after XCMS data processing. In-house MS2 database was applied for metabolite identification. Data matrix was ctr-formatted (mean-centered scaling) and pareto-scaled prior to being imported into the SIMCA V15.0.2 software package (Umetrics, Umea, Sweden). The multivariate data analysis included principal component analysis (PCA) and orthogonal partial least-squares discriminant analysis (OPLS-DA). The unsupervised PCA was implemented to demonstrate the distribution of origin data and general separation. The supervised OPLS-DA, on the other hand, was performed to obtain maximal covariance between the measured data and the response variable and validated using sevenfold cross-validation and 200 permutation tests. The validity of the OPLS-DA model was evaluated using R2Y and Q2, which were parameters for model stability and the ability to explain and predict the raw data. R2Y and Q2 values are closer to 1, suggesting a better model. The results of screening differential metabolites were visualized using a volcano plot. Euclidean distance matrix (EDM) is defined as a matrix of squared Euclidean distances between points in Euclidean space (20). It is the most commonly used distance metric in a metabolomics study (21). For comparisons of the differentially expressed metabolites between PN and PO, the EDM was calculated from the quantitative values of differential metabolites to determine the distances between each object, and then the differential metabolites were clustered by the complete linkage method and presented with a heat map. The structural information of the differential metabolites was identified according to a previous study (22). Briefly, the retention time (RT), mass-to-charge ratio (m/z), and MS/MS spectrum were rigorously matched with the authentic standards, or verified spectrums in the Mass Bank Database (http://www.massbank.jp/), Metabolite and Tandem MS Database (METLIN, http://metlin.scripps.edu/index.php), or Human Metabolome Database (HMDB, http://www.hmdb.ca/) and confirmed by our self-made m/z database. Because of the complicated nature of the untargeted metabolomics (existence of isomers and the limited accuracy of mass spectrometers) (23). All the metabolites were cautiously identified by comprehensively considering the data of m/s value, retention time, and MS/MS spectra, and finally confirmed by our self-made m/z database.

SPSS 22.0 software (SPSS Inc., Chicago, IL, USA) was used for statistical analysis. Student’s *t*-test was selected to compare the normalized integral values (clinical indices and metabolites) between PO and PN groups. The normality of data distributions was verified using the Kolmogorov–Smirnov test. All data were regarded as normally distributed because the values of Kolmogorov–Smirnov test were over 0.05. Then, homogeneity of variances was verified using the Levene’s test. The significant difference of metabolites (PO *vs.* PN) was explored. Data were presented as mean ± standard deviation. *P* < 0.05 was considered statistically significant. Metabolites that have a value of variable importance in projection (VIP) >1.0 in the OPLS-DA model and *P <*0.05 in the Student’s t-test were identified as differential metabolites (24). Because of the large number of statistical comparisons, we also calculated the Q value, which was corrected by applying the false discovery rate (FDR) to the p values using R (version 4.0.2, URL https://www.R-project.org/) (25).

### Metabolic Pathway Analysis

We searched the Kyoto Encyclopedia of Genes and Genomes (KEGG, http://www.genome.jp/kegg) (26) and MetaboAnalyst (http://www.metaboanalyst.ca/) (27) to explore the key metabolic pathways represented by the differential metabolites identified by the above experiments.

## Results

### Clinical Characteristics

**Table 1** shows the clinical characteristics in the two groups. Evidently, the participants in the PO group suffered from higher BW, BMI, waistline, and serum triglyceride level (*p* < 0.05, *vs.* women with PN). In contrast, no significant difference was found between the two groups in terms of age, height, serum TC, HDL-C, LDL-C, and serum estrogen level (**Table 1**).

**Table 1:** Clinical characteristics of the participants in two groups.

Characteristics	Perimenopausal normal (PN, n = 18)	Perimenopausal obesity (PO, n = 21)	p value
Age (year)	48.6 ± 4.3	49.0 ± 4.5	0.753
Height (cm)	153.6 ± 6.6	155.9 ± 4.4	0.215
BW (kg)	55.6 ± 5.0	67.7 ± 10.1	<0.001**
BMI (kg/m^2^)	23.6 ± 1.8	27.7 ± 3.5	<0.001**
WC (cm)	74.9 ± 3.2	90.8 ± 8.1	<0.001**
TG (mmol/L)	1.3 ± 0.7	1.9 ± 0.6	0.015*
TC (mmol/L)	5.3 ± 0.8	5.7 ± 1.5	0.268
HDL-C (mmol/L)	1.6 ± 0.3	1.6 ± 0.3	0.892
LDL-C (mmol/L)	3.1 ± 0.7	3.3 ± 1.4	0.550
Estrogen (pg/ml)	32.9 ± 36.4	32.7 ± 49.3	0.990

*p < 0.05, **p < 0.01.

### QC of the Present LC–MS Analysis

**Figure 1:**
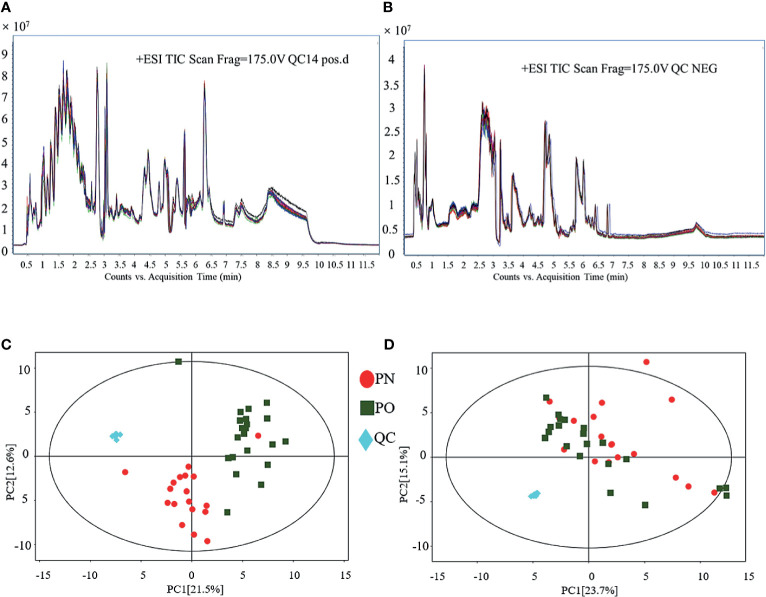
QC of LC–MS analysis. **(A)** TIC (POS); **(B)** TIC (NEG); **(C)** PCA for QC (POS); **(D)** PCA for QC (NEG).

**Figure 1** shows the results of QC of the present LC–MS analysis. The total ion chromatograms show that regardless of whether it is in the POS (**Figure 1A**) or NEG (**Figure 1B**) mode, the peak RT and area of all QC samples exhibited good overlap, indicating a satisfactory stability in the analytical system. The score scatter plot of the PCA model also presents an overlap of all QC samples, in both POS (**Figure 1C**) and the NEG (**Figure 1D**) modes, indicating a satisfactory repeatability in the analytical system, hence validating the quality of this experimental system. Then, 1482 of 1485 peaks in the POS mode and 1317 of 1344 peaks in the NEG mode were preserved after the processes of filtering, normalization, and standardization. The raw data of LC–MS analysis are provided as **Supplementary Materials** (NEG-rawdata.xlsx and POS-rawdata.xlsx).

### Multivariate Data Analysis of Serum Metabolites

**Figure 2:**
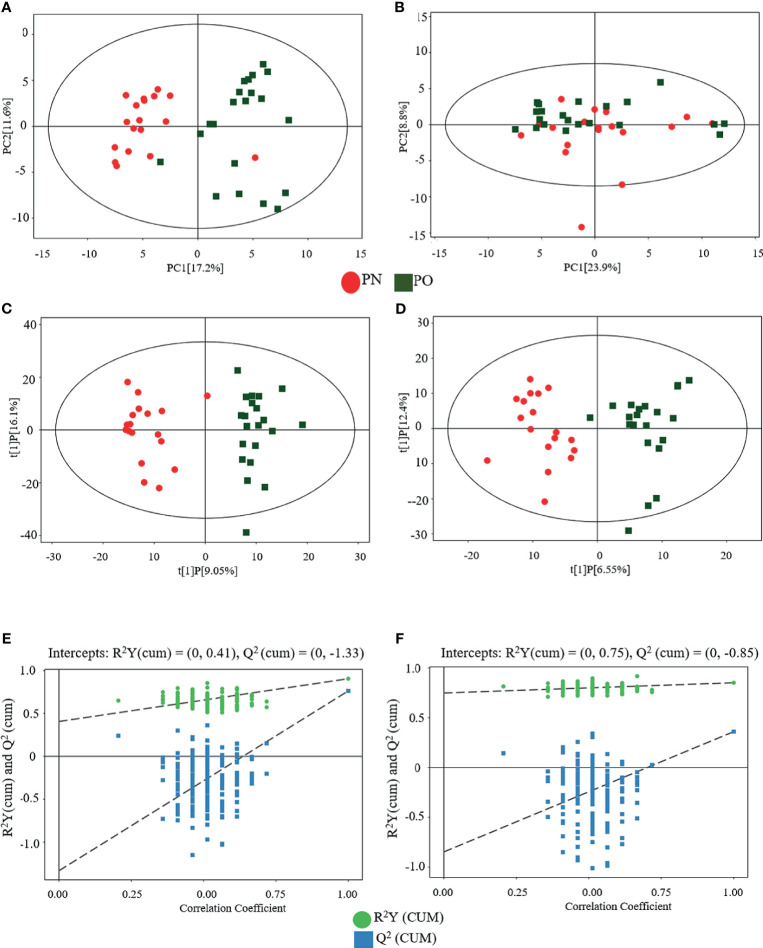
Multivariate analyses of serum metabolites. **(A)** PCA (POS); **(B)** PCA (NEG); **(C)** OPLS-DA (POS); **(D)** OPLS-DA (NEG); statistical validation of OPLS-DA model, **(E)** (POS) and **(F)** (NEG).

**Figure 2** shows the results of the multivariate data analysis of serum metabolites. As for the PCA model, the distribution of the scatter points in the PCA score plot of the UHPLC-QTOF/MS metabolic profiles of all samples was illustrated. In the POS model, the scatter points were distinctly separated between the PN and PO groups, and all samples in each score scatter plot were within the 95% Hotelling’s T-squared ellipse (**Figure 2A**). However, the clustering effect was not obvious in the NEG mode (**Figure 2B**). Then, the OPLS-DA model was constructed to discriminate the differential metabolites between the two groups. Distinct separations were presented between the PN and PO groups in this model in both POS (**Figure 2C**) and NEG (**Figure 2D**) modes. Here, the R2Y and Q2 were 0.904 and 0.762 in the POS mode and 0.851 and 0.360 in the NEG, respectively. To avoid the transition fit of the OPLS-DA mode, we subsequently conducted a permutation test. Results of the permutation test for R2Y and Q2 intercepts were 0.41 and −1.33 in the POS mode (**Figure 2E**) and 0.75 and −0.85 in the NEG mode (**Figure 2F**), demonstrating that the OPLS-DA model had no overfitting. The good quality and reliability of this OPLS-DA model were verified, meaning it was suitable to explore the differences between the two groups in this study. Then, we could perform the identification of the metabolites in the two groups.

### Identification of the Serum Metabolites in PO and PN Participants

**Figure 3:**
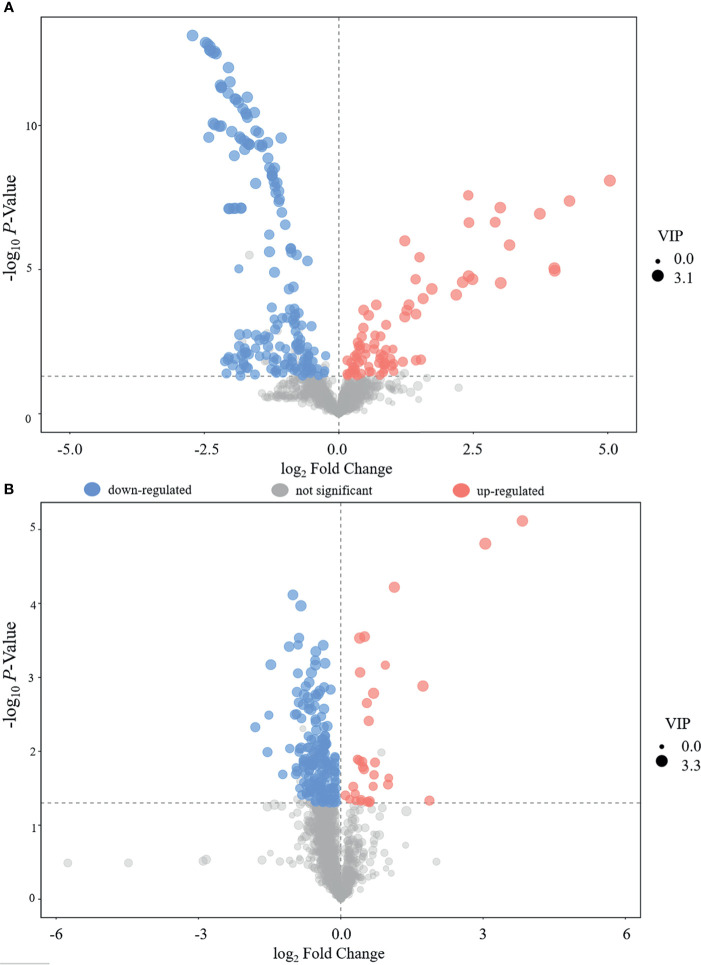
Volcano plot for the differentially expressed metabolites. **(A)** POS; **(B)** NEG.

**Figure 4:**
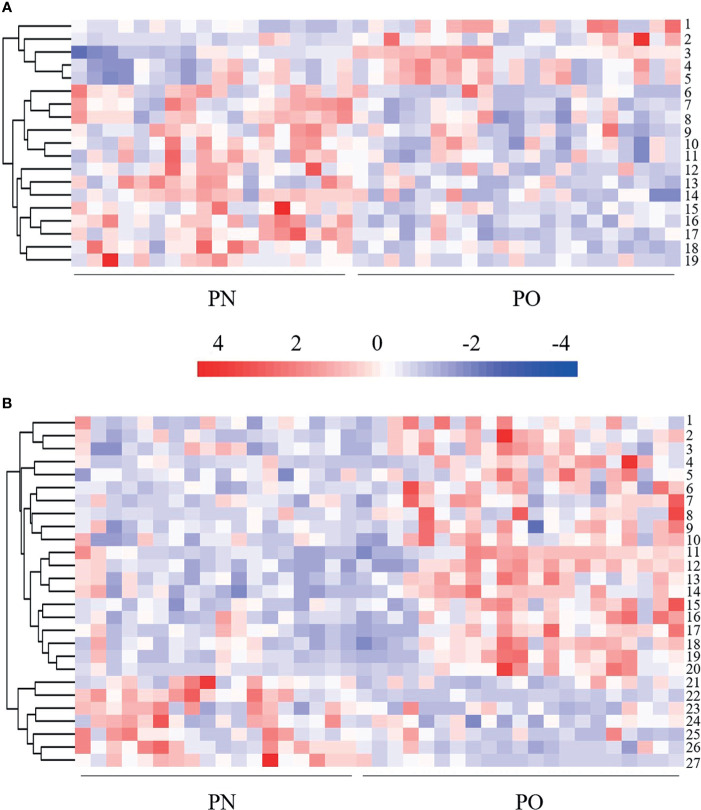
Heat map of hierarchical clustering analysis of the differentially expressed metabolites. **(A)** POS mode. 1 = d-ornithine, 2 = PC (16:0/16:0), 3 = l-arginine, 4 = pseudouridine, 5 = α-d-glucose 1-phosphate, 6 = glutaric acid, 7 = l-pipecolic acid, 8 = 3-mercapto-2-butanone; 9 = l-proline, 10 = dihydrolipoic acid, 11 = lle-Ala, 12 = Leu-Leu, 13 = myristoleic acid, 14 = xanthylic acid, 15 = Gly-Glu, 16 = *N*-acetylglutamine, 17 = His-Met, 18 = l-pyroglutamic acid, 19 = d-glucuronate. **(B)** NEG mode. 1 = indoleacetic acid, 2 = atrolactic acid, 3 = indolelactic acid, 4 = l-threonate, 5 = 5-methoxyindoleacetate, 6 = 3,4-dihydroxymandelic acid, 7 = azelaic acid, 8 = hydrocortisone 21-acetate; 9 = l-tyrosine, 10 = *N*-acetyl-l-tyrosine, 11 = dl-lactate, 12 = dihydrothymine, 13 = myristic acid, 14 = palmitic acid, 15 = l-valine, 16 = l-leucine, 17 = xanthine, 18 = l-alanine, 19 = l-glutamate, 20 = thymine, 21= *N*-acetyl-d-galactosamine, 22 = fructose 1-phosphate, 23 = 3-hydroxycapric acid, 24 = l-histidinol phosphate, 25 = 2-deoxyribose 5-phosphate, 26 = α-*N*-acetyl-l-glutamine, 27 = l-gulonic gamma-lactone.

A total of 474 metabolites were found (of those 262 in POS mode and 212 in NEG mode). Then, 340 qualitative metabolites (of those 202 in POS mode and 138 in NEG mode) were obtained by preliminary screening of all identified metabolites. Under the conditions (t-test *p* < 0.05 and OPLS-DA model VIP > 1), 46 metabolites (of those 19 in POS mode and 27 in NEG mode) were determined, which were different between the PO and PN groups. Metabolites with a significant difference were visualized through volcano plots (**Figure 3**) including carbohydrates, amino acids, organic acids, two peptides, and lipids. **Table 2** summarizes the RT, mz, VIP values, *p* values, and fold change of all these 46 metabolites. EDM was calculated using the quantitative value of a differential metabolite. We used a complete linkage method to cluster these differential metabolites and form a heat map (**Figure 4**), which shows that in the POS mode, PC(16:0/16:0), pseudouridine, l-arginine, α-d-glucose 1-phosphate, and d-ornithine were upregulated, whereas dihydrolipoate, l-proline, Ile-Ala, l-pyroglutamic acid, 3-mercapto-2-butanone, l-pipecolic acid, His-Met, xanthylic acid, N-acetylglutamine, glutaric acid, Leu-Leu, Gly-Glu, myristoleic acid, and d-glucuronate were downregulated in patients with PO. In the NEG mode, thymine, hydrocortisone 21-acetate, dihydrothymine, l-threonate, l-glutamate, atrolactic acid, xanthine, myristic acid, l-valine, azelaic acid, indoleacetic acid, l-leucine, 3,4-dihydroxymandelic acid, palmitic acid, 5-methoxyindoleacetate, dl-lactate, indolelactic acid, l-tyrosine, l-alanine, and *N*-acetyl-l-tyrosine were upregulated, whereas α-*N*-acetyl-l-glutamine, 3-hydroxycapric acid, l-histidinol phosphate, 2-deoxyribose 5-phosphate, *N*-acetyl-d-galactosamine, l-gulonic gamma-lactone, and fructose 1-phosphate were downregulated in the PO group. However, after FDR correction, only Pseudouridine, l-arginine, α-d-glucose 1-phosphate, d-Ornithine, Ile-Ala, Pyroglutamic acid, 3-Mercapto-2-butanone, l-pipecolic acid, His-Met, Xanthylic acid (XMP), Acetylglutamine, Myristoleic acid, and d-Glucuronate in POS mode, and Dihydrothymine, Myristic acid, l-valine, palmitic acid, α-*N*-acetyl-l-glutamine, l-gulonic gamma-lactone, and Fructose 1-phosphate maintained the significance of difference (**Table 2**).

**Table 2:** Differential metabolites identified in POS and NEG models.

	MS2 name	RT (min)	m/z	Relative contents		VIP	*p* value	Q value	FC
				PN	PO				
**POS mode**												
Upregulated	PC (16:0/16:0)	0.76	756.53	0.0057	0.0098	1.05	0.03	0.09	1.72
	Pseudouridine	4.72	245.08	0.1490	0.2238	1.49	<0.001	0.02	1.50
	l-arginine	8.65	175.12	15.1287	22.6698	2.05	<0.001	<0.001	1.50
	α-d-Glucose 1-phosphate	4.72	261.05	0.0402	0.0561	1.33	0.02	0.05	1.40
	d-Ornithine	8.31	133.10	0.1118	0.1537	1.11	0.01	0.03	1.38
Downregulated	Dihydrolipoate	6.30	226.08	0.9609	0.8210	1.48	0.02	0.06	0.85
	l-Proline	5.09	157.10	0.9086	0.7393	1.32	0.04	0.10	0.81
	Ile-Ala	5.98	241.10	0.0164	0.0131	1.43	0.01	0.05	0.79
	l-Pyroglutamic acid	5.31	147.08	0.0820	0.0641	1.77	<0.001	0.02	0.78
	3-Mercapto-2-butanone	4.31	209.07	0.0063	0.0048	1.68	<0.001	0.02	0.76
	l-Pipecolic acid	4.31	171.11	3.8816	2.8309	1.91	<0.001	0.01	0.73
	His-Met	5.07	304.15	0.0105	0.0077	2.20	<0.001	<0.001	0.73
	Xanthylic acid (XMP)	2.80	364.05	0.0059	0.0037	1.35	<0.001	0.01	0.63
	N-Acetylglutamine	5.07	189.09	0.1214	0.0749	2.10	<0.001	<0.001	0.62
	Glutaric acid	5.52	282.11	0.1437	0.0794	1.75	0.02	0.06	0.55
	Leu-Leu	2.62	527.32	0.0099	0.0055	1.28	0.02	0.06	0.55
	Gly-Glu	5.44	246.11	0.0375	0.0190	1.62	0.02	0.07	0.51
	Myristoleic acid	2.22	226.18	0.0238	0.0119	1.12	0.01	0.03	0.50
	d-Glucuronate	4.91	255.07	0.0249	0.0092	1.92	0.01	0.05	0.37
**NEG mode**												
Upregulated	Thymine	4.96	185.06	0.2174	0.7567	1.79	<0.001	0.08	3.48
	hydrocortisone 21-acetate	0.73	425.20	0.0458	0.0865	2.04	0.02	0.13	1.89
	Dihydrothymine	7.04	165.01	0.2288	0.4273	1.41	<0.001	0.03	1.87
	l-Threonate	6.19	195.05	0.3783	0.6984	1.06	0.01	0.11	1.85
	l-Glutamate	6.30	146.05	0.5866	1.0429	1.65	<0.001	0.07	1.78
	Atrolactic acid	1.73	165.06	0.0215	0.0333	2.53	<0.001	0.06	1.55
	Xanthine	3.45	151.03	0.4823	0.7027	1.14	0.05	0.19	1.46
	Myristic acid	0.73	227.20	0.0257	0.0372	2.35	<0.001	0.04	1.45
	l-Valine	5.56	116.07	0.0715	0.1024	2.00	<0.001	0.05	1.43
	Azelaic acid	5.42	187.10	0.0322	0.0434	1.15	0.04	0.18	1.35
	Indoleacetic acid	2.36	174.06	0.0197	0.0259	1.32	0.05	0.19	1.32
	l-Leucine	4.97	130.09	0.1478	0.1938	1.86	0.01	0.12	1.31
	3,4-Dihydroxymandelic acid	5.50	183.03	0.0306	0.0399	1.68	0.01	0.09	1.30
	Palmitic acid	0.72	255.23	0.5133	0.6636	2.28	<0.001	0.03	1.29
	5-Methoxyindoleacetate	5.57	226.04	0.0024	0.0031	1.21	0.01	0.12	1.28
	dl-lactate	3.67	89.03	14.6582	18.5742	1.39	0.01	0.10	1.27
	Indolelactic acid	2.72	204.07	0.2556	0.3184	1.96	0.04	0.18	1.25
	l-Tyrosine	4.86	180.07	1.0350	1.2788	1.43	0.02	0.12	1.24
	l-Alanine	5.56	88.04	0.5023	0.6196	1.51	0.01	0.12	1.23
	*N*-acetyl-l-tyrosine	3.53	222.08	0.0952	0.1098	1.58	0.03	0.16	1.15
Downregulated	α-*N*-acetyl-l-glutamine	4.95	187.07	0.6836	0.5187	2.65	<0.001	0.03	0.76
	3-Hydroxycapric acid	0.77	169.12	0.0178	0.0133	1.09	0.05	0.19	0.75
	l-Histidinol phosphate	4.75	280.06	0.0075	0.0054	1.81	0.02	0.12	0.72
	2-Deoxyribose 5-phosphate	2.69	213.02	2.0023	1.3580	1.58	0.05	0.19	0.68
	*N*-acetyl-d-galactosamine	1.74	242.18	0.0312	0.0205	1.25	0.05	0.19	0.66
	l-Gulonic gamma-lactone	3.97	237.06	0.0532	0.0160	2.69	<0.001	0.05	0.30
	Fructose 1-phosphate	0.37	259.03	0.3357	0.0406	3.28	<0.001	0.01	0.12

MS, mass spectrometry; m/z, mass-to-charge ratio; NEG, negative ion; PN, perimenopausal women without obesity; PO, perimenopausal women with obesity; POS, positive ion; RT, retention time; VIP, variable importance in projection.

The relative content of each metabolite was calculated by normalization of the contents with the internal standard.

### Metabolic Pathway Analysis

**Figure 5:**
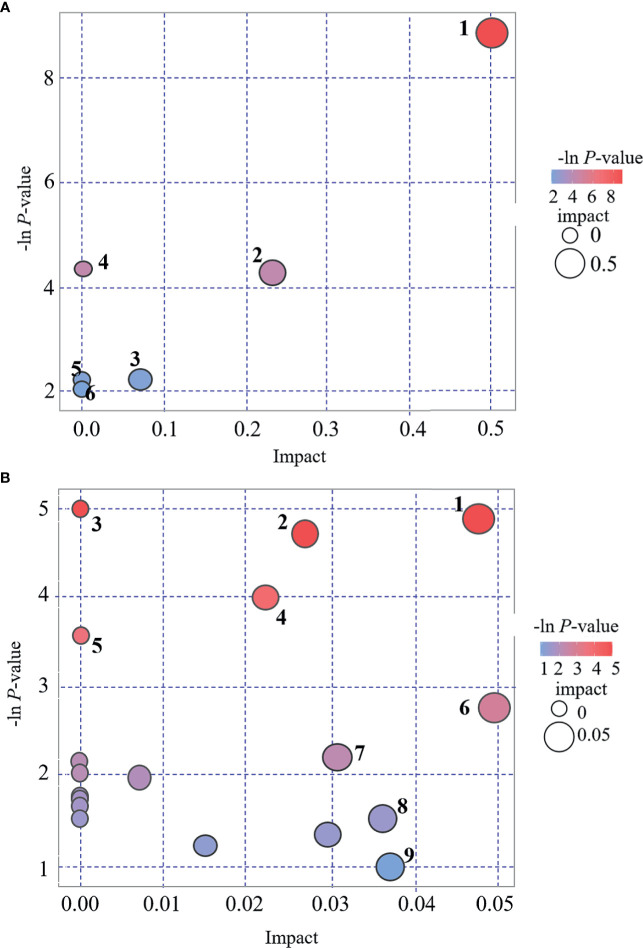
Bubble plot for pathway analysis. **(A)**: POS mode. 1 = d-arginine and d-ornithine metabolism, 2 = arginine and proline metabolism, 3 = lysine degradation, 4 = Aminoacyl-tRNA biosynthesis, 5 = fatty acid metabolism, 6 = pyrimidine metabolism. **(B)**: NEG mode. 1 = tryptophan metabolism, 2 = valine, leucine and isoleucine biosynthesis, 3 = aminoacyl-tRNA biosynthesis, 4 = Valine, leucine and isoleucine degradation, 5 = fatty acid biosynthesis, 6 = tyrosine metabolism, 7 = caffeine metabolism, 8 = fructose and mannose metabolism, 9 = purine metabolism.

KEGG and MetaboAnalyst were comprehensively used in exploring the most relevant metabolic pathways. A total of six metabolic pathways in the POS mode and 19 in the NEG were enriched (**Figure 5**), among which seven pathways exhibited a significant difference (*p* < 0.05) as follows: d-arginine and d-ornithine metabolism, arginine and proline metabolisms, aminoacyl-tRNA biosynthesis, tryptophan metabolism, valine–leucine and isoleucine biosynthesis, valine–leucine and isoleucine degradation, and fatty acid biosynthesis. Two pathways, namely, d-arginine and d-ornithine metabolism pathway and arginine and proline metabolism pathway, in the POS mode had an impact value >0.1, which were 0.50 and 0.23, respectively. Because the present study is an explorative study and for further investigation in the future, we listed all the pathways with significant difference in **Figure 5**, including those with impact value <0.1.

## Discussion

In this study, we explored the serum differential metabolites in women with PO (*vs.* women with PN) using a conventional UHPLC-QTOF/MS approach, followed by PCA and OPLS-DA. A total of 46 differential metabolites (19, POS mode; 27, NEG mode) were identified. With respect to the key metabolic pathways, a total of seven pathways were identified by KEGG and MetaboAnalyst. To the best of our knowledge, this is the first study to explore the serum metabolic characteristics in women with PO. The findings of the present study are useful to further understand the pathophysiological changes in the PO state, which can provide clues to explore/develop effective therapies in treating these pathophysiological dysfunctions and contribute in preventing the occurrence of severe obesity-related complications.

### The POS and NEG Modes in This Study

In the present study, we analyzed the metabolites in POS mode and NEG mode, respectively. POS mode and NEG mode are commonly run to analyze the metabolic profiles of samples during a traditional UHPLC-QTOF/MS approach. Many previous studies indicated that some substances were easily detected in POS mode, whereas others were easily in NEG mode (28–30). To expand the possibility of identification and determination of the metabolites in the new samples, which have never been previously tested, some authors suggested using a combination of POS and NEG modes in an explorative investigation (30). However, there is a concern that once the metabolites of positive and negative patterns are mixed together for analysis, the metabolites identified in different modes might have the same pathways, which might change the results. In the present study, we did not find any overlapped differential metabolites between these two modes (**Table 2** and **Figures 3**, **4**). Interestingly, the results of pathway analysis display that there is one pathway, namely aminoacyl-tRNA biosynthesis pathway, which was enriched in both POS and NEG models (**Figure 5**). However, this result is acceptable because the metabolites associated with the aminoacyl-tRNA biosynthesis pathway (matching amino acids with tRNAs) (31) were distributed in POS mode and NEG mode, where no overlapped amino acids were found.

### The Physiological or Pathological Meanings of the Differential Metabolites

Because of the explorative nature of the present study, we attempted to find more potential differential metabolites for further verification. Albeit there were six metabolites in POS mode and 20 metabolites in NEG mode, which could not maintain the significant difference after FDR correction (**Table 2**), they were still included in the following discussion.

#### Changes of Metabolisms of Amino Acids

As far as the amino acids, we found a significant increase of l-arginine and d-ornithine in the POS mode and l-leucine, l-valine, l-tyrosine, and *N*-acetyl-l-tyrosine in the NEG mode; a significant decrease of l-proline in the POS mode of the PO group was also noted. l-leucine and l-valine belong to branched-chain amino acids (BCAAs), whereas tyrosine belongs to the amino acid aroma (AAAs). A close association between amino acids and obesity has been noted (32). Our results agree with the previous studies that suggested higher BCAAs and AAAs in subjects with obesity (14, 32–35). The increase of arginine and ornithine suggested that the ornithine cycle might be activated and arginine utilization rate might be suppressed in women with PO. Rangel-Huerta et al. reported that the increase of arginine results from the decreased availability of arginine, which also activates the plasma asymmetric dimethylarginine level. Nevertheless, the increase of asymmetric dimethylarginine might be a marker of endothelial dysfunction (14). The key role of arginine metabolism in the PO state was also verified in our metabolic pathway analysis. We found that d-arginine and d-ornithine metabolism pathways and arginine and proline metabolism pathways in the POS mode exhibited the highest impact value (0.50 and 0.23, respectively, **Figure 5**), which seem to imply that the endothelial dysfunction might be a potentially important pathogenesis involved in women with PO. Although, by far, there is no direct evidence elucidating the intrinsic interaction between endothelial dysfunction, the increase of arginine, and onset of CVD, much indirect evidence strongly implies their close association. It is well known that CVD is one of the main causes of death of perimenopausal women (36), whereas endothelial dysfunction is an important cause of CVD (37). Moreover, arginine might be associated with endothelial dysfunction *via* a nitric oxide synthase (NOS)-related mechanism. Arginine can be catalyzed by NOS to produce NO, which has the following effects: contract and relax the muscle cells in the arteries, dilate the arteries, lower blood pressure, and thus improve blood flow. When the utilization rate of arginine decreases, the synthesis of NO also decreases, thereby resulting in the increased risk of CVD. These mechanisms were summarized as a DDAH/ADMA/NOS/NO pathway (38). A previous study suggested a significant enhancement of proline in subjects with obesity (14), whereas our study presented a converse tendency, probably due to the hindrance of proline synthesis from arginine in the PO state. In the proline–arginine metabolic pathway, synthesizing arginine to proline requires 1-pyrrolin-5-carboxylic acid synthase (P5CS) catalysis (39). Normally, P5CS exists in a circular form in the mitochondria. However, when cells are in a state of senescence and/or oxidative stress, P5CS in the mitochondria may distribute diffusely (39). Nevertheless, cell senescence is one of the most dominant characteristics in the perimenopausal period (40). Thus, we speculate that the P5CS in the cells of subjects with PO might be in a diffuse state and cannot synthesize arginine to proline, thereby causing a low serum level of proline. These mechanisms require further verification in our future investigation.

#### Changes in Lipid Metabolism

PC is one of the main phospholipids in eukaryotic cells, which can hydrolyze to produce fatty acids. It is a major component of many secretory products, such as HDL (41). PC has been reported to be significantly enhanced in postmenopausal women, which is associated with menopausal status (42). Bagheri et al. also reported higher plasma diacyl-PCs in the obesity subjects (32). All available evidence supports that the increase of PC results in the increase of fatty acids in the perimenopausal women. Excessive accumulation of fatty acids in the mitochondria leads to oxidative stress and mitochondrial dysfunction, subsequently causing many perimenopausal disorders, including obesity. Moreover, the changes of PC-related lipoproteins might be affected by hormone levels (43) as well as weight enhancement and insulin resistance (44, 45). In this study, we found that PC (16:0/16:0) is significantly upregulated in women with PO. Available literatures concerning PC (16:0/16:0) and obesity are limited. Previous analogous studies reported an increasing tendency of PC (16:0/16:0) in gestational diabetes mellitus (46) and obese pregnant women (47); however, a significant difference was not noted. Dong et al. reported a significant decrease of PC (16:0/16:0) in the myocardial tissue of diabetic cardiomyopathy rats (48). Sigruener et al. found a positive correlation between plasma PC (16:0/16:0) and mortality of patients with vascular and metabolic diseases (41). The physiological or pathological role of PC (16:0/16:0) in PO requires further investigation.

We found the upregulation of palmitic acid and myristic acid in the PO group of the NEG model, which are consistent with the previous analogous studies, which report that saturated fatty acids, such as palmitic acid and myristic acid, are increased in obese men (49) or adults (including men and women) (50) and in patients with insulin resistance (51) and T2D (52). Myristoleic acid is a desaturation product from myristic acid regulated by stearoyl-CoA desaturase (SCD) gene. In this study, we found the downregulation of myristoleic acid in subjects with PO of the POS mode. The roles of myristic acid and myristoleic acid, along with their interactions in obesity, are not fully understood. Montastier et al. reported a positive correlation between myristoleic acid and BW loss; a lower myristoleic acid means lower BW loss (53). Hence, our data presented a downregulation in the PO group, which may indicate a lower tendency of BW loss in women with PO (*vs.* PN).

#### Others

Another finding is that the serum indole lactic acid and indoleacetic acid levels are significantly upregulated in the NEG mode of women with PO. Indole lactic acid and indoleacetic acid are products of tryptophan metabolism. Their upregulation seems to imply the activation of tryptophan metabolism in the PO state. The change of tryptophan metabolism may reportedly result in a negative impact on the diversity of intestinal microflora (54), which is associated with dysfunction of the immune system and results in an increased risk of infection, malnutrition, and other functional defects (55).

#### Limitations

There are several limitations in the present study. First, we did not perform verification of the identified metabolites and pathways. For example, the increase of asymmetric dimethylarginine might be a marker of endothelial dysfunction, however, we did not examine some indicators of endothelial function in these women, although we speculate that the endothelial dysfunction might be a potentially important pathogenesis involved in women with PO. In addition, arginine can be catalyzed by NOS to produce NO; however, we did not check the NOS indicators. Indeed, rigorous verification of the metabolites and pathways, which were explored in this study, can greatly improve the strength of the evidence, which will be performed in our future investigation. Second, in addition to metabolism, many other factors, such as gene, race, diet, and lifestyle, may also contribute to the difference between PO and PN. However, in this study, we only considered age, height, and serum estrogen level and no significant difference was observed between the PO and PN groups, we therefore only performed Student’s t-test (PO *vs.* PN). More compelling methods should consider additional factors (gene, race, diet, and lifestyle, etc.) and perform multiple comparisons with post-hoc correction. Third, as an explorative study, for providing a full pathophysiological scenario of PO, we included the data from all samples, including the outlier samples shown in **Figures 1C** and **2A**; however, such data processing methods might potentially cause bias. These issues will be addressed in our future investigation.

**Figure 6:**
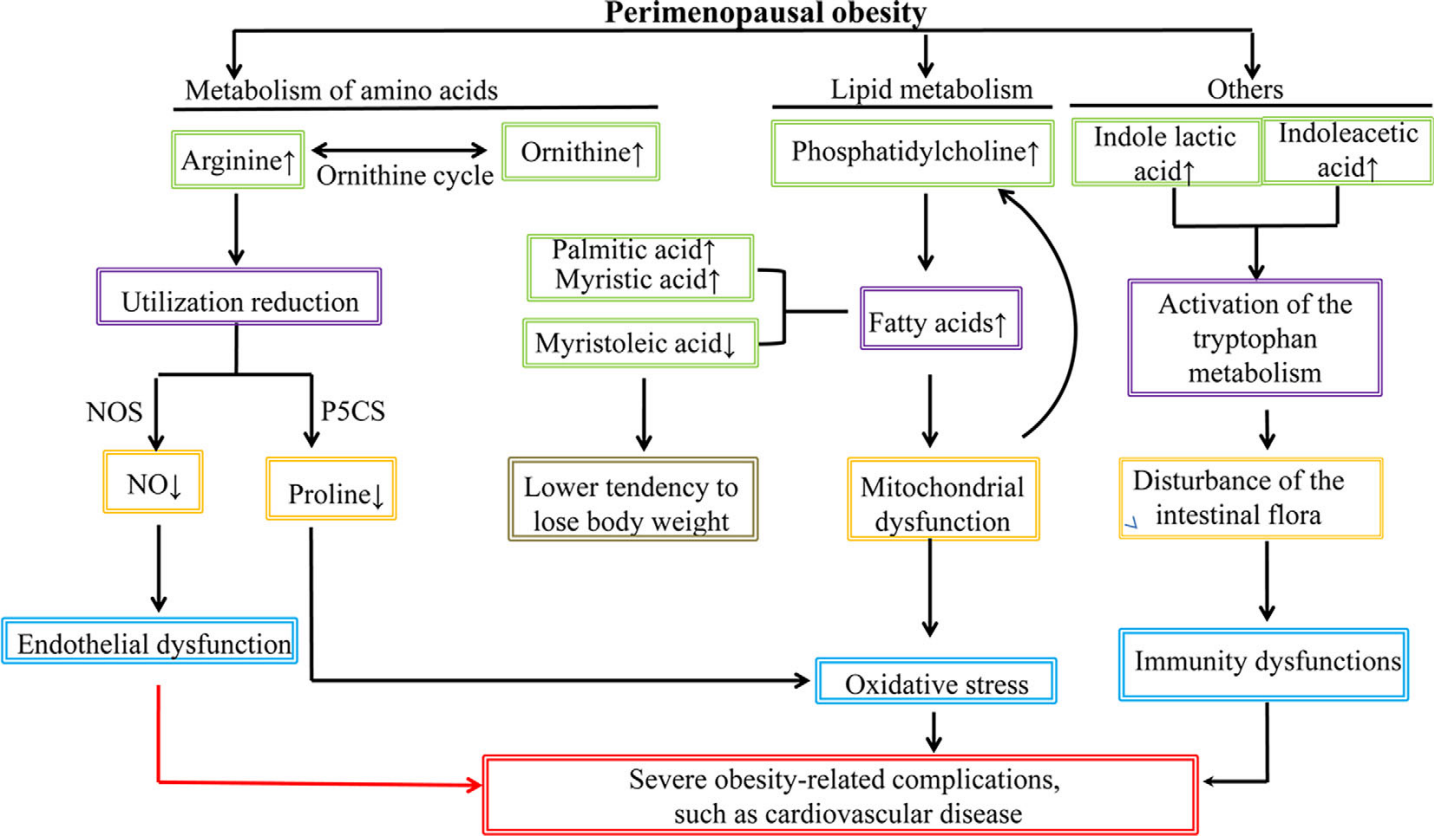
The pathophysiological scenario of perimenopausal obesity summarized in the present study.

This study explored the metabolic characteristics of women with PO by identification of the deferential metabolites using a UHPLC-QTOF/MS approach. A total of 46 differential metabolites, along with seven metabolic pathways, were identified. The differential metabolites can be divided into lipid, amino acids, carbohydrates, and organic acids. The changes of metabolisms of lipid, amino acids, and indoleacetic acid provided a pathophysiological scenario for the perimenopause women with mild obesity (**Figure 6**). Future, deeper investigation will be conducted to further uncover the pathophysiological nature of the PO, which is extremely important in preventing the incurable obesity-related diseases.

## Conclusions

A metabolomics investigation was conducted to identify the differential metabolites in perimenopausal women with mild obesity. Metabolism changes of amino acids indicated a dysfunction of the DDAH/ADMA/NOS/NO pathway, which was related to the onset of CVD; changes of lipid metabolisms indicated the tendency of BW loss and oxidative stress prevention, and the upregulation of serum indole lactic acid and indoleacetic acid levels indicated an intestinal microflora-related immunity dysfunction. Although some mechanisms of these findings require further investigation for the direct evidence, we provided a pathophysiological scenario of PO based on the metabolomic profiles explored by the UHPLC-QTOF/MS approach, which requires further verification. We believe the findings of this study are helpful for the clinicians to take measures in preventing the women with PO from developing severe incurable obesity-related complications, such as CVD.

## Data Availability Statement

The original contributions presented in the study are included in the article/**Supplementary Material**. Further inquiries can be directed to the corresponding author.

## Ethics Statement

The studies involving human participants were reviewed and approved by the ethics committee of the Fujian University of Traditional Chinese Medicine (approval number: SQ2014-007-01,study period from 2014 to 2019). The patients/participants provided their written informed consent to participate in this study.

## Author Contributions

SD and TA fetched the original ideas and designed the study, SD, QC, MC, YL, XL, SC, YC, CL, and TA performed the experiments, SD ran the statistics. SD and TA drew the figures. SD and TA wrote the first draft. TA supervised the study. All authors contributed to the article and approved the submitted version.

## Funding

This study was supported by Natural Science Foundation of Fujian Province (2020J01740). This study was also supported by the project of Fujian Province Center for Collaborative Innovation of TCM Health Management 2011 (JG2017003). This study was also supported by Fujian Education Department of China: Fujian Provincial universities’ incubation project for prominent young scientific researchers (2018). This study was also supported by the scientific research platform project of Fujian University of Traditional Chinese Medicine (X2019018-Platform). This study was also partly supported by grants from the Japan Society for the Promotion of Science (nos. 20791025, 24592157,15k10358, and 18K08991).

## Conflict of Interest

The authors declare that the research was conducted in the absence of any commercial or financial relationships that could be construed as a potential conflict of interest.

## Publisher’s Note

All claims expressed in this article are solely those of the authors and do not necessarily represent those of their affiliated organizations, or those of the publisher, the editors and the reviewers. Any product that may be evaluated in this article, or claim that may be made by its manufacturer, is not guaranteed or endorsed by the publisher.
